# Priming Picture Naming with a Semantic Task: An fMRI Investigation

**DOI:** 10.1371/journal.pone.0032809

**Published:** 2012-03-07

**Authors:** Shiree Heath, Katie McMahon, Lyndsey Nickels, Anthony Angwin, Anna MacDonald, Sophia van Hees, Kori Johnson, David Copland

**Affiliations:** 1 University of Queensland, Language Neuroscience Laboratory, Centre for Clinical Research, Brisbane, Queensland, Australia; 2 University of Queensland, Centre for Advanced Imaging, St Lucia, Queensland, Australia; 3 ARC Centre of Excellence in Cognition and its Disorders, Macquarie Centre for Cognitive Science, Macquarie University, Sydney, New South Wales, Australia; 4 University of Queensland, School of Health and Rehabilitation Sciences, St Lucia, Queensland, Australia; University Hospital La Paz, Spain

## Abstract

Prior semantic processing can enhance subsequent picture naming performance, yet the neurocognitive mechanisms underlying this effect and its longevity are unknown. This functional magnetic resonance imaging study examined whether different neurological mechanisms underlie short-term (within minutes) and long-term (within days) facilitation effects from a semantic task in healthy older adults. Both short- and long-term facilitated items were named significantly faster than unfacilitated items, with short-term items significantly faster than long-term items. Region of interest results identified decreased activity for long-term facilitated items compared to unfacilitated and short-term facilitated items in the mid-portion of the middle temporal gyrus, indicating lexical-semantic priming. Additionally, in the whole brain results, increased activity for short-term facilitated items was identified in regions previously linked to episodic memory and object recognition, including the right lingual gyrus (extending to the precuneus region) and the left inferior occipital gyrus (extending to the left fusiform region). These findings suggest that distinct neurocognitive mechanisms underlie short- and long-term facilitation of picture naming by a semantic task, with long-term effects driven by lexical-semantic priming and short-term effects by episodic memory and visual object recognition mechanisms.

## Introduction

In the treatment of word retrieval difficulties associated with post-stroke aphasia, both phonological and semantic tasks have been found to be effective [1]. While there is evidence to suggest that such training-induced improvement in naming performance can occur for some individuals [2], the underlying neurocognitive mechanisms of such improvement are not well understood. It has been argued that certain normal brain mechanisms may underpin aspects of aphasia recovery [3] and that successful treatment of word retrieval may rely on the same mechanisms that underlie priming in unimpaired naming [4]. However, there has been no direct examination of the mechanisms underpinning the facilitation of naming using commonly employed semantic treatment tasks, in unimpaired individuals. The present study, therefore, aimed to examine in healthy older adults the effects associated with a typical semantic facilitation technique on subsequent picture naming performance using functional magnetic resonance imaging (fMRI). This is vital not only to elucidate the mechanisms underpinning aphasia treatment success, but also to further our understanding of the mechanisms of facilitation in the unimpaired brain.

Picture naming is a basic linguistic skill requiring the integration of multiple component processes. Importantly, behavioral research has shown that picture naming can be facilitated with experience. For instance, the act of naming a picture once speeds the subsequent naming of that picture, even up to 48 weeks following a single exposure [5], [6]. This performance enhancement has been referred to as repetition priming [7]. Various accounts have been put forward to explain repetition priming and its positive effect on performance. One proposal is that priming effects involve a modification of representations at certain levels of cognitive processing during first presentation, so that recognition and retrieval processes are enhanced on subsequent presentations [8], [9]. A second proposal is that priming may be mediated by episodic retrieval [6], [9], whereby a memory trace is generated on prime presentation and it is retrieval of this memory on target presentation which is the source of facilitation, rather than any difference in the accessibility or retrieval of knowledge from within the word production system.

However, identifying the locus of priming is difficult, as the facilitation of picture naming may occur through any of the multiple stages involved in word production. During the conceptual-semantic stage the features and meaning of a picture must be activated from within an individual's semantic system [10]. The correct representation must then be converted into lexical word-level knowledge. This occurs at the lexical-semantic stage, where stored conceptual-semantic knowledge enables selection of the appropriate lexical entry. It is this abstract lexical unit which is then given phonetic form during the phonological stage [11], where the phonological properties of the word are brought together and prepared for articulation. Connections must therefore exist between the processing levels, enabling a mapping operation linking word meaning and word form [12]. The present study utilized fMRI to investigate for the first time in healthy older adults the underlying neurocognitive effects of a semantic task upon subsequent picture naming.

Previous research has identified a large network of perisylvian neural regions which support the processes involved in word production and these areas are distributed throughout the frontal, parietal and temporal lobes. Further, it appears that the different component processes of naming engage specific brain regions [13], [14]. The middle and inferior temporal gyri, the anterior and mid-portions of the inferior frontal gyrus, and the angular gyrus of the parietal lobe have been linked to semantic processing [15]–[20]. In contrast, phonological processing has been associated with the superior temporal gyrus, the posterior portion of the inferior frontal gyrus and the supramarginal gyrus of the parietal lobe [13], [15], [17], [20]–[22]. However, previous studies have either simply compared areas of neural activation engaged by semantic or phonological tasks to some sort of baseline activity [23], or used the same task on both prime and target presentations [24]–[26]. Additionally, the majority of these studies have investigated effects over relatively short periods, often less than 30 seconds. Few have utilized a specific facilitatory prime task directed at one of the component processes involved in naming to investigate the longevity of any effects. The present study investigates the facilitatory effects of a semantically focused task on subsequent picture naming over a period of several minutes (in the short-term), as well as over a period of days (in the long-term).

Some studies have investigated repetition priming in picture naming over long timeframes and found experience-related changes in neural activity, reflecting less effort being required to encode and identify a repeated object name [27], [28]. However, these studies have generally relied on covert verbal responses [29] and have not specifically targeted the semantic component of word production processes. Other neuroimaging research has attempted to explore more closely the direct contribution of semantic priming by examining the behavioral and cortical activation associated with the repetition of a semantically related task. These studies, also utilizing the same task at both prime and target presentations, have shown that repeated retrieval of semantic information can result in a decrease in response latency and a decrease of neural activity in regions linked to semantic processing [30]–[32]. This research has revealed that the facilitation resulting from a semantic task may indeed result in long-lasting facilitatory effects. For example Meister, Buelte, Sparing and Boroojerdi [33] used a semantic decision task (e.g. a judgment regarding whether a written word could be classified as representing an abstract or concrete concept) to examine long-term semantic priming effects on repeated performance of the same task. Long-term conceptual priming was associated with altered activity in regions known to be involved in semantic processing (e.g. the left inferior prefrontal cortex) over a period of three days. However, the authors drew limited conclusions regarding the level of processing responsible for the facilitation effects identified and simply noted that similar regions have been reported in semantic priming studies using shorter retention intervals, indicative of similar mechanisms being responsible for both short- and long-term priming effects [30], [33], [34]. Their study is also limited by the fact that the semantic task required participants to make a decision regarding written words and, therefore, the orthographic form of each stimulus item was present. The presence of the word form means that both phonological and lexical-semantic processes could have been engaged during performance of the task. In this regard, previous studies could be advanced in two important respects. Firstly, instead of looking at the facilitatory effects of a semantic task upon subsequent performance of the same task, it may be more meaningful to investigate how a semantic task at prime presentation contributes to the facilitation of subsequent picture naming. Secondly, the semantic component of word production may be targeted by utilizing a selective task which requires lexical-semantic processing, but which does not include presentation of the word form and, therefore, has less emphasis on phonological processing.

Although framed as semantic, the locus of the facilitatory effects resulting from such tasks remains a matter of debate in healthy individuals and patient populations [1], [2], [9], [35], [36]. Further complicating matters is the suggestion that possible sources of facilitation may operate over different timeframes. As previously mentioned, some studies in healthy controls have shown robust repetition priming effects over long time periods (lasting up to several days) following semantically related tasks [28], [33], [37]. Early behavioral studies investigating semantic priming using a lexical decision task, however, have proposed that facilitation targeting the lexical-semantic level has a short duration, with transient effects declining after a few seconds or a few intervening items [38], [39]. It is apparent that competing evidence exists for both short-term and long-term semantic facilitation effects and it is unclear what cognitive mechanisms are contributing to the longevity of effects. One explanation is that longer lasting facilitation is due to a strengthening of the mapping from the lexical-semantic to the phonological level [9]. Support for this proposal was provided by Wheeldon and Monsell [9] who investigated the effects on picture naming of previously producing the name of an item in response to a definition over the short-term (10 to 35 seconds) or long-term (6 to 12 minutes). Evidence of strong and reliable facilitation over both time frames suggested a robust change in the cognitive pathway shared by producing a name in response to a definition and picture naming (i.e. between semantics and phonology). In contrast, a second experiment by the same authors [9] found that prior production of a homophone of the target item (a word that shares pronunciation, but not meaning, e.g., flour, flower) was insufficient to facilitate subsequent picture naming (6 to 12 minutes later). Clearly, the phonological word form needed to be associated with the meaning of the target item for facilitation to occur, providing further weight to their argument that long-term repetition priming effects can be attributed to a strengthening of the links between the semantic and phonological levels of processing.

It is, however, unclear at what level of processing the facilitation of picture naming using a semantic task is having an effect, and how long lasting any resultant effects may be. The present fMRI study sought to elucidate the time course and neurocognitive substrates underlying the facilitation of word retrieval by a semantic task. We examined the facilitation effects on picture naming over a period of minutes (in the short-term) and also over a longer period of days (in the long-term), which could be indicative of a more enduring change in processing [28], [40]. Importantly, the three main naming conditions of interest were presented in a single scanning session. The study adds significantly to a limited body of research by utilizing for the first time a semantically-focused task, which does not involve the phonological word form, to facilitate subsequent overt picture naming over different time frames in healthy older adults. Rather than identifying overall changes to the processing associated with repeated picture naming, this unique methodology seeks to determine how a targeted semantic facilitation technique, performed in the absence of the phonological form of the target, affects the subsequent processing of items during successful naming. Our findings will test competing accounts regarding whether short-term or long-term facilitatory effects are possible from a semantic task. In terms of the mechanisms responsible, we hypothesized that if facilitation is having an effect primarily at the lexical-semantic level, the regions engaged would include the anterior and mid-portions of the inferior frontal gyrus, the middle and inferior temporal gyri, and the angular gyrus of the parietal lobe. Alternatively, if indeed the primary mechanism underlying facilitation from a semantic task is a strengthening of the connections between the lexical-semantic and phonological levels, then we hypothesized that facilitation would involve regions linked to both semantic and phonological processing. These phonological regions, in addition to areas known to be associated with semantic processing, would include the posterior portion of the inferior frontal gyrus, the superior temporal gyrus and the supramarginal gyrus of the parietal lobe.

## Methods

### Ethics Statement

Ethical approval was obtained from the University of Queensland Medical Research Ethics Committee in accordance with the Declaration of Helsinki and written consent obtained from each participant.

### Participants

Nineteen healthy older adults participated in the study. The data from one participant was removed from subsequent analyses due to a high percentage of delayed responses (36.4% of correct responses >1500 ms). The average age of the remaining 18 (10 female) older adults was 56.2 years (SD = 10.4, range 38 to 74 years). The average tertiary educational level of participants was 16.6 years (SD = 3.0, range 12 to 22 years). Participants received no direct financial benefit from involvement in the study, but were reimbursed for travel costs. All were native speakers of English, were right handed, and had normal or corrected to normal vision. Exclusionary criteria included significant hearing loss (identified by pure tone audiometry screening), a history of alcohol abuse, mental illness, any other neurological disease or disorder, as well as the presence of any metal objects within the body, or other contraindications for MRI. Participants were also tested for visual acuity, screened for cognitive impairment with the Mini-Mental State Examination [41] and for depression with the Geriatric Depression Scale [42].

### Stimuli

The 60 experimental stimuli and 12 practice stimuli items were obtained from the Hemera digital photographic database (Hemera Photo-Objects, Hemera, Hull, Canada), as well as other royalty-free digital stock photographs. Items were classified across ten broad semantic categories (including objects, animals, food, clothing, people, vehicles, tools, places, natural phenomena, and body/animal parts), distributed evenly across conditions. All images were photographs of approximately the same size (no larger than 500×420 pixels) with a consistent white background canvas size (600×600 pixels). Images were grey-scaled, with an average luminance of 223.68 (cd/m^2^) (SD = 19.84, range 151.44 and 253.67). Frequency counts were obtained from the CELEX lexical database [43] and age of acquisition norms from Morrison, Chappell and Ellis [44]. Associated imageability ratings were obtained from the MRC psycholinguistic database [45]. The 60 stimulus items were then divided into three sets, with 20 items in each set and each set assigned to a different main condition of interest – either unfacilitated, short-term facilitated, or long-term facilitated. Assignment of sets to conditions was counterbalanced across all participants. Sets (including filler items) were matched on the basis of reaction time, frequency, age of acquisition, number of phonemes, number of syllables, percentage name agreement and imageability. Additionally, the Edinburgh Associative Thesaurus [46] was utilized to ensure no stimuli items within a set were first associates of each other. The auditory stimuli for semantic questions were spoken by a female voice and digitally recorded at 44100 Hz, mono, 32 bit, in a sound-proof recording studio.

### Procedure

**Figure 1:**
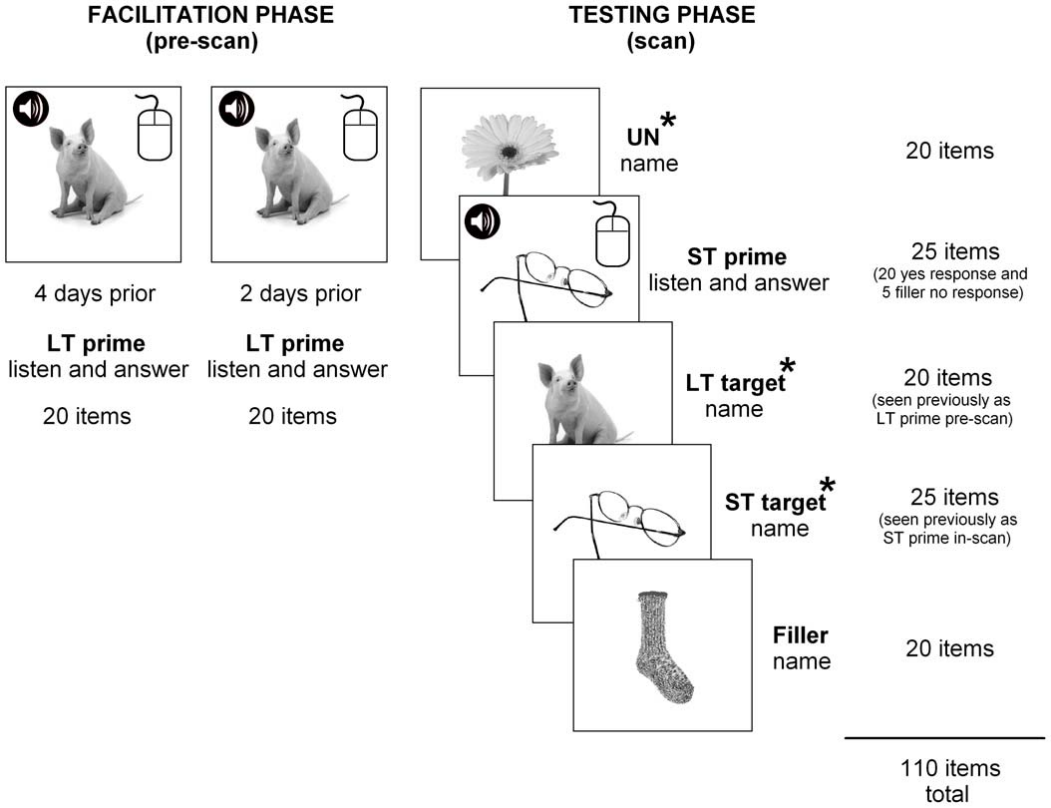
A summary of randomized stimuli presentation. ***Facilitation phase***: one set of 20 pictures were presented three times on two separate occasions (six times total), simultaneously with a semantic auditory question, for yes/no response by computer mouse button (LT prime). ***Testing phase (during scan)***: the long-term facilitated set were presented again for naming (LT target); a set of 20 pictures were presented twice - once as a prime along with an auditory question for yes/no response (ST prime) and then presented again (6 to 12 trials later) for naming (ST target); and one set of 20 unfacilitated pictures was also presented once for naming (UN), along with an additional set of 20 unfacilitated non-critical fillers. * indicates the three main naming conditions of interest – unfacilitated, long-term facilitated and short-term facilitated.

The study utilized a single independent factor of facilitation (short-term facilitated, long-term facilitated, or unfacilitated), with picture naming accuracy, reaction times and neural activity as measured by fMRI as primary dependent variables of interest. The experiment was conducted over two weeks and involved two main phases (see Figure 1). In the first phase (facilitation phase) each participant was required to complete two facilitation sessions, no more than three days apart, during which one set of 20 stimuli was presented three times, each time in a different random order. The behavioral task used in the facilitation phase of the study was created using E-Prime (version 1.1) (Psychology Software Tools, Pittsburg, PA). A single facilitation trial consisted of a fixation point (+) displayed for 1,500 ms, followed by a target picture for a period of 3,000 ms. Each target picture was presented simultaneously with an auditory question regarding the semantic properties of the item. The 20 target stimuli items required a positive response and an additional 10 filler items, also presented three times randomly, required a negative response and were interspersed to ensure unpredictability of response type. Participants were required to respond either yes, or no, by computer mouse button press. Both the hand used to respond and the mouse button used to represent yes and no was counterbalanced across participants. Additionally, the questions did not contain the relevant target item. For example, for the target picture ‘DOG’, the question was “Does it bark?” as opposed to “Does a dog bark?”. In other words, participants were not presented with, or required to produce, the phonological word form during facilitation. Additionally, no other target item was included in the question (e.g., the question for the picture ‘KEY’ did not include the words “lock” or “door” if these were also target items). Semantic questions were based upon Garrard, Lambon Ralph, Hodges and Patterson's [47] classification types and took the form of either sensory (e.g., “Is it sharp?”), functional (e.g., “Is it eaten?”) or encyclopaedic (e.g., “Is it found in the ocean?”), distributed evenly across conditions. Upon completion of both facilitation sessions, participants had been randomly presented with each picture stimulus item from this one set of 20 items, together with its corresponding auditory semantic question, a total of six times. As previously mentioned, the type of task employed in the current study is similar to those used in clinical treatments of word retrieval deficits in aphasia. Word finding treatments are generally administered intensively, with multiple presentations of a small number of items. In fact, studies suggest that several repetitions are required for long-lasting effects on naming ability [48], and it is for this reason that our long-term facilitated condition involved a total of six presentations of stimuli. This aspect of the methodology does, however, limit the comparability of the current study to other repetition priming or facilitation-based research that generally employs only a single prior exposure of stimuli. No feedback was given regarding accuracy of responses during facilitation sessions.

The second phase (testing phase) involved an fMRI scanning session, with all three sets of stimuli presented for naming, including an additional 20 items as non-critical fillers. Microsoft Visual Basic 6.0 (Microsoft Corporation, Redmond, WA) was used to create the behavioral task for the testing phase. Naming responses were digitally recorded (sampling rate 11 kHz) with an optical single channel noise cancelling microphone (FOMRI, Optoacoustics Ltd., Or-Yehuda, Israel). The experimental stimuli were enlarged and back-projected onto a luminous white screen which participants viewed through a mirror mounted on the head coil and subtended approximately 10 degrees of visual arc. fMRI sessions were conducted in three runs, with two runs of 35 individual trials and one run of 40 trials, resulting in 110 trials in total. A single trial lasted 14.7 s and consisted of a 250 ms period of blank screen, followed by a target picture displayed for a period of 3 seconds. This was followed by a blank screen for 9.45 s, then a fixation point (+) displayed for 2 s marked the commencement of the following trial. The long-term facilitated set of items, which had previously been presented during the facilitation sessions, was presented again in the scanner to investigate any long-term facilitation effects (with no more than two days between the final facilitation session and scan). The short-term facilitated set of items was presented twice within the scanner (in different random order): once as a prime, along with an auditory semantic question for a yes/no response by participants (via mouse button), and then presented again as a target for naming to investigate any short-term facilitation effects (within a lag of 6 to 12 trials, average 10 trials, over a period of no more than 3 minutes). The set of unfacilitated items was also presented once within the scanner as a baseline for comparison purposes. To ensure the semantic properties associated with each target item were engaged, critical short-term facilitated items required a positive response to semantic questions. An additional five filler questions requiring a no response were randomly interspersed within the short-term prime condition, and subsequently presented again for naming, so that the pattern of yes and no responses was inconsistent. Stimuli were presented pseudo-randomly in blocks of five trials per condition (long-term facilitated condition, short-term facilitated prime and target conditions, and unfacilitated condition), interspersed across the course of the scanning session. Stimuli were blocked to ensure participants were aware of what task was required for each item and to minimize any effects of constant task switching. In this regard, at the commencement of each trial block of five items, either the word “Name” (for critical and filler target items) or the word “Answer” (for short-term facilitated prime items and filler questions requiring a no response) was displayed in the centre of the screen to indicate task instructions.

### Image Acquisition

Images were acquired using a whole body 4-Tesla Bruker MedSpec scanning system (Bruker Medical, Ettingen, Germany). A transverse electromagnetic head coil was utilized [49] to enhance imaging resolution at a high field strength. Gradient-echo, echo planar images (GE-EPI) (matrix size of 64×64; repetition time (TR) 2100 ms; echo time (TE) 30 ms; 90° flip angle; field of view 230 mm) with an interleaved gradient acquisition sequence were acquired in 36 axial planes with in-plane resolution of 3.6 mm and slice thickness of 3 mm (0.6 mm gap). A behavioral interleaved gradient design was employed to obtain minimal scanner noise during picture presentation and response time (4.2 s). In this regard, only slice gradients were applied during the critical interval, with radiofrequency intact to maintain steady state magnetization [50]. For the following 10.5 s in which the blank screen (8.5 s) and fixation point (2 s) were displayed, image acquisition occurred to capture the BOLD response for that naming trial. This design was utilized to avoid artefacts associated with head movement during overt responses, to establish accurate reaction times, as well as enable recording of responses [50], [51]. A total of 625 GE-EPI volumes were acquired over three runs, with the first five volumes (the first 10.5 s) in each run discarded to allow magnetization to reach steady state. A PSF (point-spread function) mapping sequence was acquired prior to GE-EPI acquisitions, allowing the distortion in geometry and intensity to be corrected in the time series data [52]. A three-dimensional T_1_ weighted MP-RAGE (magnetization-prepared rapid gradient-echo) was acquired within the same session (matrix size of 256×256; TR 2200 msec; TE 2.99 msec; inversion time (TI) 900 msec; 9° flip angle; resolution 1.0×1.0×1.0 mm; FOV 256 mm).

### Data Processing

Naming trials which elicited no response, or an incorrect response, from participants were excluded (5.04% of responses). Data for the short-term facilitation prime trials in the testing phase were also excluded, as these trials did not involve naming. We also removed the filler prime items requiring a no response and their subsequent target presentations. Images were processed and analysed using Statistical Parametric Mapping (Version 5) software (SPM5, Wellcome Department of Cognitive Neurology, London, UK) with MATLAB 2009a (The MathWorks Inc., Natick, MA). The image time series were first realigned during spatial preprocessing using rigid body motion correction with INRIAlign [53]. The mean EPI image generated from the realigned series for each participant was coregistered with the T_1_ image acquired in the same session. The T_1_ image was then segmented and normalized to the standard Montreal Neurological Institute (MNI) [54] atlas T_1_ weighted template. These transformations were applied to the realigned EPI time series. Normalized volumes (3×3×3 mm^3^) were then spatially smoothed using an 8 mm full-width half-maximum Gaussian kernel. Due to the partial collection of hemodynamic response function, a factor of the behavioral interleaved design, a general linear model (GLM) for the fMRI time series was constructed using finite impulse response functions. Age was included as a covariate in the GLM and the onsets and durations were chosen to reflect the expected BOLD peak.

### Data Analysis

Hypothesis-driven regions of interest (ROIs) were determined based upon the findings of various language-related meta-analyses, including Vigneau et al. [20]. A total of nine left hemisphere spherical ROIs (of 6 mm radius) were defined (MNI coordinates) using the MarsBaR region of interest toolbox [55] for SPM5. Three ROIS were identified as having been previously associated with phonological processing, including the posterior region of the inferior frontal gyrus (pars opercularis) (−54, 12, 20), the posterior portion of the superior temporal gyrus (−50, −38, 12) and the supramarginal gyrus of the parietal lobe (−42, −52, 37). In addition, six ROIs associated with semantic processing were selected within the anterior (pars orbitalis) and mid (pars triangularis) portions of the inferior frontal gyrus (−37, 31, −9; −43, 20, 4), the anterior superior temporal gyrus (−56, −13, −5), the mid-section of the middle temporal gyrus (−59, −37, 1), the posterior inferior temporal gyrus (−46, −55, −7), and the angular gyrus of the parietal lobe (−45, −68, 26). The temporal cortex ROIs chosen were within Indefrey and Levelt's [13] y-coordinate delineation of anterior, middle and posterior temporal regions. A GLM, repeated measures ANOVA was conducted to compare main effects and results are reported at *p*<0.05. A whole brain analysis was also conducted, with automated anatomical labeling software [56] used to identify the neuroanatomical location of peak maxima. A Monte Carlo estimation procedure was conducted with 10,000 simulations using 3dClustSim (implemented in Analysis of Functional Neuroimages, National Institute of Mental Health, Bethesda, MD) [57]. A height threshold of *p*<0.001 was adopted in conjunction with a cluster threshold of *p*<0.05 estimated for the whole brain, resulting in a corrected cluster size of at least 23 contiguous voxels.

## Results

### Behavioral results

**Figure 2:**
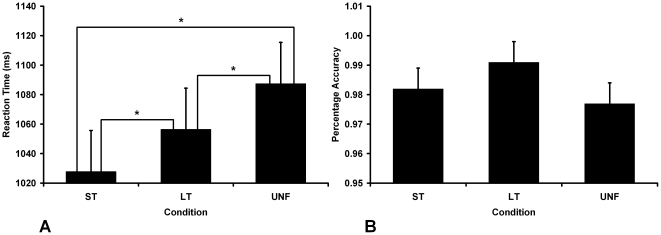
Facilitation effects in behavioral data. **A.** Mean reaction times and standard errors for each condition. **B.** Mean percentage accuracy for each condition. Significant differences were found between all conditions for naming latency (*p*<0.05). No significant differences were identified between conditions for percentage accuracy. Error bars indicate standard error mean.

Naming latencies and accuracy data from the testing phase of the study are shown in Figure 2. An initial linear mixed model analysis, with subject as a random factor and condition as a fixed factor, was conducted on all behavioral data which included age as a covariate. The reaction time data analyses were conducted on correct responses, with times below 200 ms and above 1,500 ms removed (8.61% of correct responses) and the accuracy analyses conducted on all trials. Participants' age did not interact significantly with condition for either reaction time (*F*_2,922_ = 1.784, *p* = 0.169) or accuracy (*F*_2,1058_ = 0.222, *p* = 0.801). Further analysis of the reaction time data showed a main effect for condition (*F*_2,924_ = 11.886, *p*<0.001), and post-hoc pairwise comparisons identified significant differences between all conditions (*p*<0.05). In this regard, both short- and long-term facilitated items were named significantly faster than unfacilitated items, with short-term items significantly faster than long-term items. No main effect of condition was found for accuracy upon further analysis, with the mean percentage accuracy being above 97% for all conditions. There was a trend for long-term facilitated items to be named most accurately, with short-term items named more accurately than unfacilitated items.

### Imaging results

**Figure 3:**
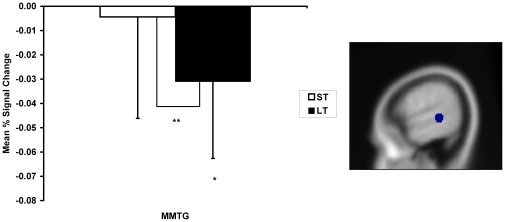
Region of interest analysis. Figure displays the a priori defined region of interest (the mid-portion of the middle temporal gyrus), within which significant differences in activation (*p*<0.05) were identified for two key contrasts. Bar graph indicates relative mean percentage BOLD signal change as a function of facilitation, compared to the unfacilitated condition. * indicates significant changes in mean signal intensity compared to the unfacilitated baseline (*p*<0.05) and ** indicates a significant difference between long-term (LT) and short-term (ST) facilitated conditions. Error bars indicate standard error mean.

**Figure 4:**
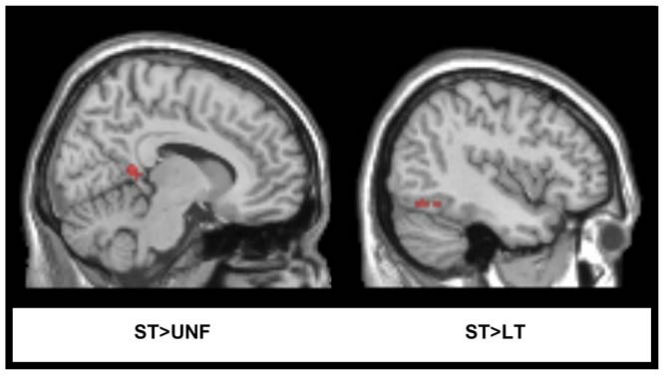
Whole brain analysis. Regions showing significant BOLD response for relevant contrasts of interest (at *p*<0.001) for clusters greater than 23 contiguous voxels (as per 3dClustSim minimum cluster size) displayed on the SPM5 T_1_ canonical brain in a series of sagittal slices. Parameter estimates: ST>UNF = 0.04±0.01 and ST>LT = 0.0185±0.0055.

Of the nine left hemisphere cortical ROIs examined, only a single region showed significant differences in activation for two key contrasts of interest (see Figure 3). Long-term facilitated items showed decreased activity in the mid-portion of the middle temporal gyrus (MMTG) when compared to both unfacilitated (*p* = 0.032) and short-term facilitated items (*p* = 0.010). A subsequent whole brain analysis (set out in Table 1 and Figure 4) was then conducted. The whole brain results identified an increase in activity for short-term facilitated items within certain language-related regions: in the right lingual gyrus (extending to the precuneus region) when compared to unfacilitated items and in the left inferior occipital gyrus (extending to the left fusiform gyrus) when compared to long-term facilitated items. Changes in activity for main contrasts of interest were also identified in the primary motor and somatosensory cortices: in the left precentral gyrus greater activity was identified for long-term facilitated items than for short-term facilitated items, and a decrease for short-term facilitated items in the right postcentral gyrus and for long-term facilitated items in the right precentral gyrus when compared to unfacilitated items. Modulation of activity in motor cortices has been associated with visuo-spatial attentional mechanisms and is often identified during language tasks [58]. For example, reduced premotor activity for primed words relative to unprimed words has previously been observed, consistent with more efficient motor programming [59]. However, the following discussion focuses primarily on findings in traditional language-related regions.

**Table 1:** Whole brain results.

Contrast Description and Anatomical Label	Volume	x	y	z	Z-score
Short-Term>Unfacilitated					
right lingual gyrus extending into precuneus	29	9	−42	6	4.20
Short-Term>Long-Term					
left inferior occipital gyrus extending into left fusiform gyrus	36	−39	−69	−9	4.19
Long-Term>Short-Term					
left precentral gyrus	28	−30	−9	42	3.98
Unfacilitated>Short-Term					
right postcentral gyrus	24	30	−42	69	4.15
Unfacilitated>Long-Term					
right precentral gyrus	28	57	−12	45	3.45

MNI coordinates of peak activation from whole brain analysis (*p*<0.001) for clusters greater than 23 contiguous voxels (as per 3dClustSim minimum cluster size).

## Discussion

This study investigated the short- and long-term facilitatory effects of a semantically-focused task upon subsequent overt picture naming and its neural correlates in healthy older adults. The major findings of the current study suggest that distinct mechanisms underlie short- and long-term facilitation of naming using a task requiring lexical-semantic processing, but which does not include the phonological word form. More specifically, long-term effects appeared to be driven by lexical-semantic priming and short-term effects by episodic memory and visual object recognition mechanisms. We now set out a detailed discussion of these major findings.

### Repetition suppression for long-term facilitation

Many studies utilizing object repetition have found a decrease in magnitude of fMRI signal for repeated items [25], [28]. This relative decrease in cortical activity following stimulus repetition is referred to as “repetition suppression” and is thought to reflect greater processing efficiency [60]–[62]. Based on our stated hypotheses and previous behavioral findings [9], if long-lasting facilitation is due to an enduring change in the strength of the links between the two processing components, a decrease in activity in regions previously associated with both lexical-semantic and phonological processing may be expected. However, our ROI results identified decreased activation for long-term facilitated items compared to both unfacilitated and short-term facilitated items, only in the MMTG. The middle temporal gyrus has previously been linked to lexical-semantic processing [20], [63]–[65] including lexical selection [13], [16], [59], [66], [67]. Therefore, the finding of a repetition suppression effect in the MMTG for long-term facilitated items could reflect a form of durable and additive lexical priming, with more efficient selection of representations at the lexical-semantic level of processing for these items previously presented multiple times over the long-term.

If lexical priming is driving the long-term facilitation of a semantic task upon subsequent picture naming, then one might expect to find a similar effect for short-term facilitated items. One possible explanation for a repetition suppression effect in the MMTG being limited to long-term items that have been facilitated multiple times is that this lexical priming effect may be additive. Picture naming studies directly comparing the effects of a single exposure to multiple repetitions over time have similarly identified activation decreases within occipitotemporal regions, as objects become more familiar with experience [27], [60]. In the current investigation, an additive effect would not be expected for unfacilitated items and would not be as pronounced for short-term facilitated items seen only once previously, resulting in no significant effect for these items. This cumulative effect also fits with research identifying practice-related changes in neural activity after multiple exposures to a variety of language tasks, which is thought to result in increasingly more efficient processing through repeated use [68], [69]. This interpretation suggests the need to reconsider the proposal that long-term facilitation only arises from a strengthening of the connections between the lexical-semantic and phonological levels of processing [1], [9].

### Repetition enhancement for short-term facilitation

An increase in activity for short-term items was identified when compared to both unfacilitated and long-term facilitated items in the whole brain results. A “repetition enhancement” effect is often present when additional processing is required [27], [60] and previous studies have identified such an effect, with increased activity upon repeated stimulus presentation [28], [70], [71]. However, in the present study the task required for each of the three main conditions of interest was overt picture naming. As such, there should not have been additional processing specifically required for short-term facilitated items. This repetition enhancement effect for short-term facilitated items was found within the right lingual gyrus (extending into the precuneus region) when compared to unfacilitated items. While many studies investigating visual object processing have found extensive occipital activation regardless of task [72], the bilateral lingual gyri have been linked to perceptual identification processes and episodic encoding [73]. Portions of the precuneus have also been linked to mental imagery processes and episodic memory retrieval [74]. Episodic memory retrieval and recollection is one of the mechanisms put forward to account for the repetition priming effect [74], [75]. The role of episodic memory in picture naming is presumed to involve the ability to form a record of past meaningful experiences, particularly those involving semantic associations and concepts [76], and is therefore closely linked to semantic processing. Although the picture naming task utilized during the current study did not require any explicit recall of previous presentations of stimuli, it may be that episodic encoding or object recognition systems engaged during the earlier performance of a semantic task were enhanced upon subsequent naming of short-term facilitated items.

Increased activity for short-term items when compared to long-term facilitated items was also identified within the left inferior occipital gyrus (extending into the left fusiform gyrus). The inferior occipital gyrus and fusiform gyrus form part of the ventral visual processing pathway, linked to visual association and object recognition [77], [78]. The fusiform gyrus, in particular, has been reliably activated in semantic processing studies [76] and, given its proximity to regions linked to object perception, some authors have proposed that portions of the fusiform region play a specific role in retrieval of information regarding the visual attributes of objects [76], [79], [80]. Increased activation for short-term facilitated items in this region may, therefore, be due to active visual recognition of the prime picture stimuli presented a few minutes previously. It appears that a recent single exposure to picture stimuli has resulted in repetition enhancement effects upon subsequent naming in brain regions involved in visual processing and object recognition. These findings suggest that short-term facilitation may be primarily driven by the brain mechanisms underpinning automatic recognition or recall of visuo-perceptual representations associated with object priming, rather than lexical-semantic mechanisms.

It is noteworthy that the results of the current study did not identify any modulation of activity within the inferior frontal gyrus for contrasts of interest. The left inferior frontal region has been linked to semantic processing and, in particular, to the selection of semantic information from amongst competing alternatives [81]. Complex tasks involving high-level semantic selection demands, such as word generation [68], semantic judgment [31] and monitoring [18], are likely to result in inferior frontal gyrus involvement. Conversely, studies investigating implicit semantic priming effects using a simple lexical-decision task do not always identify differences in activation within the inferior frontal gyrus [59], [82]. As previously mentioned, in the present study the task required for each of the three main conditions was overt picture naming, with no high-level integrative processing required. Therefore, a lack of significant differences in inferior frontal activation is not entirely unexpected.

Finally, although this study did not aim to examine age-related changes in processing, effects of aging on priming were examined in order to exclude any potential confound. The literature investigating priming effects in aging is limited and while early behavioral studies found a reduction in priming effects in very elderly participants [83], [84], recent research claims that priming is age-invariant [85]–[87]. Aligned with these more recent findings, the present study found no age-related effects in this cohort of healthy older controls. Despite this, it should be noted that the results of the current study are not able to be generalized to the wider population and comparison to other studies may be limited due to differences in age of participants.

### Conclusions

Taken together, the results of the current study indicate that two distinct mechanisms underlie the facilitatory effects of prior semantic processing upon subsequent picture naming, and that these mechanisms operate over different timeframes. Our finding of a repetition suppression effect for long-term facilitated items in the MMTG suggests that the locus of this enduring, and possibly additive, facilitation is at the semantic and/or lexical-semantic level of processing. Repeated performance of a semantic task has enabled more efficient activation and selection of lexical-semantic information, which in turn speeds retrieval of the lexical item and naming. The short-term facilitatory effects stemming from performance of a semantic task, however, appear to be consistent with episodic memory and object recognition mechanisms associated with repetition enhancement effects in neural regions linked to object recognition.
